# The Wertheim-Watkins Operation for Cystocele

**Published:** 1911-10

**Authors:** R. R. Kime

**Affiliations:** Atlanta, Ga.


### THE WERTHEIM-WATKINS OPERATION FOR CYSTOCELE.

By R. R. Kime, M. D., Atlanta, Ga.

This operation is an evolution from colpoceliotomy or vaginal celiotomy.

Bandler states:—"Sanger first theoretically (1888) recommended colpoceliotomy. Durssen first performed the operation by accident (1890) tearing through the peritoneal fold in doing a vaginal fixation of the uterus. The same accident occurred to Zweifel and Fritsch. Duhrasen began opening the peritoneum purposely through the vagina anteriorly in 1892. Mackenrodt was also a pioneer in doing anterior vaginal celiotomy for other work than hysterectomy."

Following these, Wertheim,-Watkins and Goffe evolved an operation for cystocele which in selected cases gives the best results of any operative procedure yet devised for this condition. The operation is contra-indicated in women that are to become pregnant again. In such a lighter and simpler operation must be performed that will not interfere in future pregnancies.

The majority of such lighter operations are practical failures because the denudation is not carried deep enough and over a broad enough surface. Then mucous membrane is simply brought together in place of closing wound with tiers of superimposed sutures, forming a solid basis for bladder.

It is fortunate that many of these cases are past the menopause or middle age and have borne several children, so that if need be they can be rendered sterile without removal of ovaries and an operation done that will give permanent relief.

While I am not an advocate of rendering women sterile without just cause, yet I feel that a woman past the age of forty years, who has borne several children, and is suffering with a marked cystocele with its associate conditions is entitled to relief at the hands of the medical profession when it can be best done by such an operation. This is one of the operations in which vaginal celiotomy is given preference to abdominal section. At the time this operation is done conservative work on tubes, ovaries and, if necessary, round ligaments can be done, even a resection of a part of body of uterus, if such a procedure is deemed necessary. The operation is preceded by dilating cervix and curetting uterus followed by amputation of cervix and repair of perineum when indicated.

In repair of the perineum I prefer the flapsplitting synthetic perneotomy as most certain to get best results.

I regret I overlooked preparing this paper until I received notice of this meeting, then it was too late to have drawings made to illustrate the operation, so a brief description of operation mus suffice.

The patient properly prepared, in dorsal position, disinfection of parts completed, preliminary disinfection being previously performed.

An inverted T shaped incision in anterior vaginal wall gives best space for work.

The transverse incision at cervico-vaginal junction, best made with scissors, vaginal mucosa caught up at center with two artery forceps gives purchase for holding up vaginal flap, separating from cervix, then up anterior wall of uterus, with gauze and blunt dissection, lifting up bladder, taking care not to pass through its wall, separate bladder from uterus until peritoneum is reached, then it must be caught up and incised, entering abdominal cavity, the incision and dissection may be widened by introducing the index fingers of both hands, widening same, taking care not to injure ureters. The vaginal wall is separated from bladder either by introducing scissors and separating blades or safest, by splitting vaginal mucosa longitudinally between forceps up to crest of cystocele and by careful dissection separate it from bladder. This being accomplished the excess of flaps cut off the next step is anteversion of uterus.

A narrow long blade retractor facilitates the work both in incising the peritoneum and in elevating the bladder for anteverting the uterus, which is readily done by catching vulsella in cervix, pushing it downward, backwards and upwards into hollow of sacrum, then catch anterior surface of uterus with sharp claw retractors by an over and over motion bring body of uterus forward and outward as practiced by Ochsner and Mayo.

In this position conservative work on tubes and ovaries can be done or patient made sterile by cutting fallopian tube from corner of uterus by a V-shaped incision, closing up same, tieing tube and stitching end of tube to uterine wall, closing over exposed raw surfaces. If tube is diseased it should be removed.

Best not to stitch bladder peritoneum to uterus, it will fall back over posterior wall of fundus, especially when it fills with urine, if stitched sutures might tear out or a dead space be formed for collection of fluids.

Three or four sutures are now introduced through anterior vaginal mucous flap, commencing at upper angle of longitudinal incision, including anterior wall of uterus as high toward fundus as desired, then out through the other side of mucous flap. The anterior surface of uterus between ligatures is scarified or scraped, fundus is now slightly elevated, sutures pulled out, together and a protecting suture now introduced (Bandler), at upper angle through mucosa and anterior wall of uterus and tied, to prevent bladder wall slipping down in further manipulations. The fixation sutures being tied, the remainder of longitudinal incision is closed with continuous or interrupted catgut, the transverse incision is now closed, a part of it in a longitudinal manner if desired. The fixation sutures may be chromic catgut or plain catgut hardened in alcohol and iodine, some use two stay sutures of silk, remainder catgut.

If cervix is to be amputated, it should be done before stay sutures are tied, the perineal repair being done last when it is needed, which is usually necessary if we expect the best results from the operation.

In cases where the uterus is large and somewhat prolapsed but not seriously diseased, it should be resected and so much of it removed as is necessary to give proper adjustment for support of bladder and vaginal wall.

Those interested in the technique of the operation can find excellent illustrations in Bandler's Vaginal Celiotomy and in article by Watkins in Surgery, Gynecology and Obstetrics, May, 1909—Vol. VIII, page 471.

